# Altered visual entrainment in patients with Alzheimer’s disease: magnetoencephalography evidence

**DOI:** 10.1093/braincomms/fcac198

**Published:** 2022-08-01

**Authors:** Seth D Springer, Alex I Wiesman, Pamela E May, Mikki Schantell, Hallie J Johnson, Madelyn P Willett, Camilo A Castelblanco, Jacob A Eastman, Nicholas J Christopher-Hayes, Sara L Wolfson, Craig M Johnson, Daniel L Murman, Tony W Wilson

**Affiliations:** Institute for Human Neuroscience, Boys Town National Research Hospital, Omaha, NE 68010, USA; College of Medicine, University of Nebraska Medical Center, Omaha, NE 68198, USA; College of Medicine, University of Nebraska Medical Center, Omaha, NE 68198, USA; Montreal Neurological Institute, McGill University, Montreal, QC H3A 2B4, Canada; College of Medicine, University of Nebraska Medical Center, Omaha, NE 68198, USA; Institute for Human Neuroscience, Boys Town National Research Hospital, Omaha, NE 68010, USA; College of Medicine, University of Nebraska Medical Center, Omaha, NE 68198, USA; Institute for Human Neuroscience, Boys Town National Research Hospital, Omaha, NE 68010, USA; Institute for Human Neuroscience, Boys Town National Research Hospital, Omaha, NE 68010, USA; Institute for Human Neuroscience, Boys Town National Research Hospital, Omaha, NE 68010, USA; Institute for Human Neuroscience, Boys Town National Research Hospital, Omaha, NE 68010, USA; Institute for Human Neuroscience, Boys Town National Research Hospital, Omaha, NE 68010, USA; Center for Mind and Brain, University of California Davis, Davis, CA 95616, USA; College of Medicine, University of Nebraska Medical Center, Omaha, NE 68198, USA; College of Medicine, University of Nebraska Medical Center, Omaha, NE 68198, USA; College of Medicine, University of Nebraska Medical Center, Omaha, NE 68198, USA; Memory Disorders & Behavioral Neurology Program, University of Nebraska Medical Center, Omaha, NE 68010, USA; Institute for Human Neuroscience, Boys Town National Research Hospital, Omaha, NE 68010, USA; College of Medicine, University of Nebraska Medical Center, Omaha, NE 68198, USA; Department of Pharmacology & Neuroscience, Creighton University, Omaha, NE 68178, USA

**Keywords:** magnetoencephalography, Alzheimer’s, dementia, entrainment, vision

## Abstract

Recent research has indicated that rhythmic visual entrainment may be useful in clearing pathological protein deposits in the central nervous system of mouse models of Alzheimer’s disease. However, visual entrainment studies in human patients with Alzheimer’s disease are rare, and as such the degree to which these patients exhibit aberrations in the neural tracking of rhythmic visual stimuli is unknown. To fill this gap, we recorded magnetoencephalography during a 15 Hz visual entrainment paradigm in amyloid-positive patients on the Alzheimer’s disease spectrum and compared their neural responses to a demographically matched group of biomarker-negative healthy controls. Magnetoencephalography data were imaged using a beamformer and virtual sensor data were extracted from the peak visual entrainment responses. Our results indicated that, relative to healthy controls, participants on the Alzheimer’s disease spectrum exhibited significantly stronger 15 Hz entrainment in primary visual cortices relative to a pre-stimulus baseline period. However, the two groups exhibited comparable absolute levels of neural entrainment, and higher absolute levels of entertainment predicted greater Mini-mental Status Examination scores, such that those patients whose absolute entrainment amplitude was closer to the level seen in controls had better cognitive function. In addition, 15 Hz periodic activity, but not aperiodic activity, during the pre-stimulus baseline period was significantly decreased in patients on the Alzheimer’s disease spectrum. This pattern of results indicates that patients on the Alzheimer’s disease spectrum exhibited increased visual entrainment to rhythmic stimuli and that this increase is likely compensatory in nature. More broadly, these results show that visual entrainment is altered in patients with Alzheimer’s disease and should be further examined in future studies, as changes in the capacity to entrain visual stimuli may prove useful as a marker of Alzheimer’s disease progression.

## Teaser

Springer *et al*. report differences in neural processing of rhythmic visual stimuli in Alzheimer’s disease patients relative to healthy controls. These results, along with correlations between visual responses and cognitive performance, suggest a compensatory effect, such that patients with Alzheimer’s disease require stronger cortical responses to encode similar visual information.

## Graphical Abstract

**Graphical abstract:**
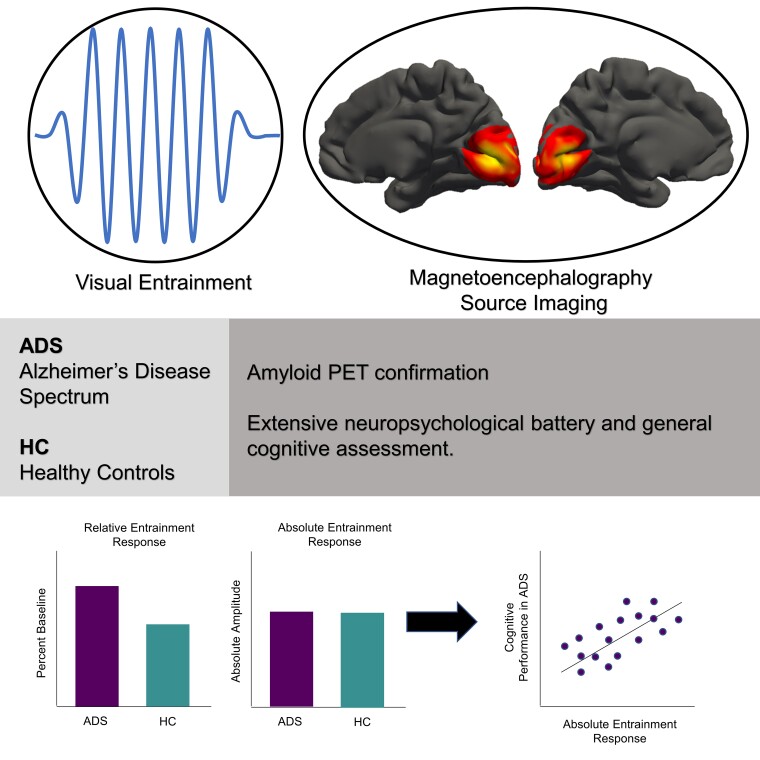

## Introduction

Alzheimer’s disease progression is widely recognized to exist along a continuum (i.e. spectrum), starting with pre-clinical stages in which symptoms are undetectable, and progressing through mild cognitive impairment (MCI) until the ultimate diagnosis of Alzheimer’s type dementia is reached.^1–3^ This continuum is marked by a progressive accumulation of amyloid-β ‘plaques’ and ‘tangles’ of phosphorylated tau, alongside widespread neurodegeneration and alterations to neuronal function.^4^ Throughout the Alzheimer’s disease progression, the primary visual cortices are commonly considered to be relatively spared, with no disease-related changes in visual acuity.^5–7^ However, numerous studies of visual evoked potentials in patients on the Alzheimer’s disease spectrum have suggested neural aberrations. The vast majority of this work has shown early visual response amplitude decreases and latency increases,^8–14^ with the exception of the frequency-resolved analysis of Yener *et al.*^15^ which revealed increased theta band amplitude responses in patients with Alzheimer’s disease during basic visual processing. Furthermore, in recent years, groundbreaking studies in mouse models of Alzheimer’s disease have shown that sustained rhythmic visual entrainment in the gamma band leads to reductions in amyloid-β (Aβ) and hyperphosphorylated tau in the primary visual cortices, hippocampus, and prefrontal cortex, and partially rescues cognitive function.^16–18^ These effects are thought to be mediated by increased microglial co-localization with Aβ,^16–18^ indicating that the increased neural activity induced by visual entrainment might boost local amyloid phagocytic activity, which is known to be deficient in patients with Alzheimer’s disease.^19^ In light of these mouse model findings, there is now increased interest in the role of rhythmic neuronal activity in patients on the Alzheimer’s disease spectrum, particularly in the visual cortices.

Visual entrainment occurs when neurons in the visual cortices synchronize their firing to the frequency of a flickering visual stimulus. This response is distinct from transient visual responses, which reflect the firing of neurons after the presentation of a single visual stimulus.^20^ Clinically, visual entrainment paradigms have proven useful in the characterization of patients with neurological and psychiatric disorders (e.g. migraines and schizophrenia),^21–24^ and their counterparts in the auditory domain have shown similar promise (e.g. in autism and schizophrenia).^25,26^ Despite extant clinical applications of visual entrainment paradigms, to our knowledge, the fidelity of neural tracking of rhythmic visual stimuli in patients on the Alzheimer’s disease spectrum is virtually unstudied, even at the frequencies known to elicit the most robust entrainment responses in healthy adults (i.e. 14–16 Hz).^27,28^ Given the emerging evidence of amyloid reductions in mouse models of Alzheimer’s disease, identifying potential differences in the cortical tracking of rhythmic visual stimuli in patients on the Alzheimer’s disease spectrum is critical and could provide both key insights on disease progression and the necessary data for translating this paradigm into humans.

In this study, we utilize the high spatio-temporal precision of magnetoencephalography (MEG) to identify differences in the entrainment of neural populations to rhythmic visual stimulation between patients on the Alzheimer’s disease spectrum and healthy controls. Based on the few previous neuroimaging studies of visual processing in patients on the Alzheimer’s disease spectrum, we expected these patients to exhibit significantly weaker visual entrainment responses. Furthermore, supporting the notion that these visual entrainment responses reflect a meaningful correlate of clinical/neuropsychological pathology in Alzheimer’s disease, we also predicted that they would scale with the degree of cognitive impairment in these individuals.

## Methods and materials

### Participants

Forty-four patients with amnestic mild cognitive impairment (aMCI) or mild probable Alzheimer’s disease were enrolled in the Alzheimer’s disease spectrum group for this study. Classification as aMCI or mild probable Alzheimer’s disease was made based on established guidelines^29^ by a fellowship-trained neurologist specializing in memory disorders, and all 38 patients included in the final Alzheimer’s disease spectrum group were identified as being amyloid-positive by whole-brain amyloid PET (collected for the purposes of this study). Of the six patients who were excluded from the study, one had COVID-19 related safety concerns, one had an incidental finding on structural imaging that warranted exclusion, and four exhibited amyloid-negativity on PET. The remaining 38 biomarker-confirmed patients on the Alzheimer’s disease spectrum were compared with a group of 20 adult healthy controls (19 amyloid-negative and one without amyloid biomarkers). Of note, the 19 amyloid-negative healthy control participants were recruited based on their previous enrolment in an unrelated clinical trial of an anti-amyloid drug in cognitively healthy older adults. These participants were discovered to be amyloid-negative during the screening process from this clinical trial and were excluded from participation prior to the intervention. These participants did not report cognitive disturbances, which was confirmed by our own detailed neuropsychological assessments (see *Methods: Neuropsychological Testing* and Table 1) and were not being seen in clinic for amnestic disturbances. The two groups were matched on all key demographics except age (i.e. the Alzheimer’s disease spectrum group was slightly younger on average), however, inclusion of age in the statistical models resulted in no meaningful changes to the statistical significance or interpretation of the results. Exclusionary criteria included any medical illness affecting CNS function, neurological or psychiatric disorder (other than Alzheimer’s disease/aMCI), history of head trauma, standard exclusionary criteria for MEG studies (e.g. dental braces, metal implants, and/or any type of ferromagnetic implanted material), current substance abuse and moderate or severe depression (Geriatric Depression Scale ≥ 10). Written informed consent was obtained from each participant (and for patients, from their informant as well) following a description of the study. In cases where capacity to consent was questionable, informed assent was obtained from the research participant, in addition to informed consent from a legally authorized representative. The Institutional Review Board at the University of Nebraska Medical Center reviewed and approved this study.

**Table 1:** Group-wise demographic and neuropsychological profiles

Clinical characteristics	Alzheimer's disease spectrum (*n* = 38)	Controls (*n* = 20)	*P*-value
Age (years)	69.21 (6.91)	72.70 (4.73)	0.048
Sex (% female)	47	60	0.416
Education (years)	15.50 (2.72)	16.60 (2.87)	0.156
MMSE	24.16 (3.77)	29.40 (0.88)	<0.001
MoCA*	19.18 (4.87)	27.43 (1.99)	<0.001
Learning	–2.14 (0.88)	0.60 (0.76)	<0.001
Memory	–2.28 (0.70)	0.33 (0.55)	<0.001
Language	–1.04 (1.01)	0.18 (0.76)	<0.001
Processing speed	–0.90 (1.42)	0.66 (0.83)	<0.001
Attention	–0.77 (1.06)	0.53 (0.60)	<0.001

*n = 48 (controls = 14; ADS = 34). MMSE = Mini-Mental Status Examination, MoCA = Montreal Cognitive Assessment.

### Neuropsychological testing

Participants performed a battery of neuropsychological assessments, which were developed in collaboration with a clinical neuropsychologist specializing in memory disorders and were focused on five cognitive domains known to be affected in Alzheimer’s disease: *verbal memory* [Wechsler Memory Scale (WMS-IV) Logical Memory II Delayed Recall and Recognition;^30^ Hopkins Verbal Learning Test-Revised [HVLT-R] Delayed Recall and Recognition Discriminability Index^31^), *learning* (WMS-IV Logical Memory I Recall;^30^ HVLT-R Learning Trials 1–3^31^), *attention and executive function* (Wechsler Adult Intelligence Scale [WAIS-IV] Digit Span Forward, Backward and Sequencing;^32^ Trail Making Test Part B^33^), *language* (Boston Naming Test;^33^ Controlled Oral Word Association Test/Phonemic Verbel Fluency;^33^ Animals/Semantic Verbal Fluency^33^), and *processing speed* (WAIS-IV Coding;^32^ Trial Making Test Part A^33^). Neuropsychological assessment raw scores for each participant were converted to demographically adjusted *z*-scores (i.e. age, education, and so on) using published normative data.^31–34^

General cognitive processes were examined using the Montreal Cognitive Assessment (MoCA)^35^ and the Mini-Mental Status Examination (MMSE).^36^ In addition, the Functional Activities Questionnaire^37^ was used to measure instrumental activities of daily living with the assistance of an informant for patients on the Alzheimer’s disease spectrum.

### Florbetapir ^18^F PET

Combined PET/CT data using ^18^F-florbetapir (Amyvid™, Eli Lilly) and a GE Discovery MI digital scanner (Waukesha, WI) were collected following the standard procedures described by the Society of Nuclear Medicine and Molecular Imaging (3D acquisition; single intravenous slow-bolus <10 mL; dose = 370 MBq; waiting period = 30–50 min; acquisition = 10 min).^38^ Images were attenuation corrected using the CT data, reconstructed in MIMNeuro (slice thinkness = 2 mm),^39^ converted to voxel standardized uptake values based on body weight and normalized into MNI space. Each scan was read by a fellowship-trained neuroradiologist blinded to group assignment and assessed as being ‘amyloid-positive’ or ‘amyloid-negative’ using established clinical criteria.^39^ At this stage, patients who were amyloid-negative were excluded from the Alzheimer’s disease spectrum group.

### Experimental paradigm

During the MEG recording, participants sat in a non-magnetic chair within a magnetically shielded room and were instructed to fixate on an entrainment stimulus that flickered at a rate of 15 times per second (Hz). The stimulus was a small white circle 3.8 cm in diameter that was presented centrally on a black background and subtended an approximate visual angle of 1.83°. The duration of each flicker-train was 1.5 s and the inter-stimulus interval was randomly jittered between 2.5 and 3.0 s. The entrainment stimuli were presented in E-Prime (version 2.0; Psychology Software Tools, Pittsburgh, PA), and the stimulation frequency was verified using a fast Fourier transform of data from a fibre-optic photodiode attached to the presentation screen while the experimental paradigm was displayed. Each participant completed 120 trials, which resulted in a total recording time of about 9 min. To ensure compliance, an MEG technologist continuously monitored participants during data acquisition via real-time audio–video feeds from inside the shielded room. Participants whose neural data or physical demeanour suggested that they had become drowsy or disengaged were indicated as such in our logs and asked to repeat the recording.

### Structural MRI acquisition, processing and coregistration with MEG data

Preceding MEG analysis, four coils were attached to the subject’s head and localized, together with the three fiducial points and at least 100 scalp surface points, with a 3-D digitizer (FASTRAK 3SF0002, Polhemus Navigator Sciences, Colchester, VT, USA). Once the participants were positioned for MEG recording, an electric current with a unique frequency label (e.g. 321 Hz) was fed to each of the coils. This induced a measurable magnetic field and allowed each coil to be localized in reference to the sensors throughout the recording session. Since coil locations were also known in head coordinates, all MEG measurements could be transformed into a common coordinate system. With this coordinate system, each participant’s MEG data were co-registered with their structural T_1_-weighted MRI prior to source space analysis using the fiducials and all digitized scalp surface points in Brain Electrical Source Analysis (BESA) MRI (Version 2.0). Structural MRI data were aligned parallel to the anterior and posterior commissures and transformed into standardized (i.e. Talairach) space using direct 3D-spline interpolation. Following source analysis (i.e. beamforming), each subject’s functional MEG images were also transformed into standardized space using the transform that was previously applied to the structural MRI volume and spatially resampled to enable group-wise statistical comparisons.

### MEG data acquisition, preprocessing and imaging

Neuromagnetic responses were sampled at 1 kHz using an Elekta/MEGIN MEG system (Helsinki, Finland) with 306 sensors (102 magnetometers and 204 planar gradiometers). Importantly, only the 204 gradiometers were used for analysis, given that our task was designed to elicit neural activity in primary visual cortices relatively close to the sensor array, for which the sensitivity profile of planar gradiometers is ideal. MEG data from each participant were individually corrected for head motion and subjected to noise reduction using the signal-space separation method with a temporal extension (correlation limit: 0.950; correlation window duration: 6 s).^40,41^ Cardiac and ocular artefacts (blinks and eye movements) were removed from the data using signal-space projection, and this correction was accounted for during source analysis.^42^ The continuous magnetic time series was divided into epochs of 3800 ms duration, with the onset of the cue defined as 0 ms and the baseline defined as the preceding 600 ms (−600 to 0 ms). Epochs containing artefacts were rejected based on a fixed threshold method that was set per participant and supplemented with visual inspection. Briefly, in MEG, the raw signal amplitude is strongly affected by the distance between the brain and the MEG sensor array, as the magnetic field strength falls off sharply as the distance from the current source increases. To account for this source of variance across participants, as well as actual variance in neural response amplitude, we used an individually determined threshold based on the signal distribution for both signal amplitude and gradient to reject artefacts. The average amplitude cut-off threshold for patients on the Alzheimer’s disease spectrum was 1004.11 fT (SD = 356.02), whereas the average threshold for controls was 1050.75 fT (SD = 332.05); this did not differ by group (*t*_56_ = 0.485, *P* = 0.63). The average gradient cut-off threshold for patients on the Alzheimer’s disease spectrum was 181.16 fT/s (SD = 126.60) and the same for controls was 171.60 fT/s (SD = 93.38); again, this did not differ by group (*t*_56_ = –0.297, *P* = 0.767). On average, after artefact rejection, patients on the Alzheimer’s disease spectrum had 103.29 (SD = 9.26) trials and controls had 101.40 (SD = 7.76) trials remaining. There was no significant difference in the number of accepted trials per group (*t*_56_ = –0.779; *P* = 0.44).

Artefact-free epochs were transformed into the time–frequency domain using complex demodulation,^43–45^ with a frequency-step of 0.5 Hz and a time-step of 100 ms between 4 and 50 Hz, using a 1 Hz lowpass finite impulse response (FIR) filter. These sensor-level data were then averaged across all trials to generate time–frequency plots of mean spectral density per sensor and normalized with respect to the mean baseline power (i.e. –600 to 0 ms). The specific time–frequency window used for subsequent source imaging was determined using a stringent statistical analysis involving non-parametric permutation testing of the sensor-level spectrograms across all participants and the entire array of gradiometers.^46–48^

Each participant’s MEG data were co-registered to their individual high-resolution structural MRI. Cortical responses were then imaged to a spatial grid of 4.0 × 4.0 × 4.0 mm using the dynamic imaging of coherent sources beamformer.^49^ Following convention, the source power from these images were normalized per voxel using a pre-stimulus noise period (i.e. baseline) of equal duration and bandwidth.^50^ All MEG data preprocessing and imaging used the BESA 7.0 software. To assess the anatomical basis of the responses identified through the sensor-level analysis, 3D maps were normalized into Talairach space and then averaged across all participants. To investigate the neural differences in visual processing between participants on the Alzheimer’s disease spectrum and healthy controls, virtual sensors (i.e. voxel time series data) were extracted from each participant’s MEG data. Specifically, we identified the voxel with the strongest entrainment response in the grand average image (i.e. across all participants) and computed virtual sensors for that location by applying the sensor weighting matrix derived from the forward solution to the preprocessed signal vector, which yielded a time series for the specific voxel in source space. These virtual sensor time series were then transformed into the time–frequency domain using the same complex demodulation procedure as the sensor-level time–frequency decomposition. From these time–frequency virtual sensor data, the envelope of spectral power was computed for the frequency range used in the beamforming analysis (i.e. 14.5–15.5 Hz). For each participant, the average baseline activity was also derived by averaging the absolute amplitude time series data across the baseline period (i.e. –600 to 0 ms). To derive the relative time series, the absolute amplitude time series was normalized using the same –600 to 0 ms baseline period. Estimates of the relative and absolute entrainment response amplitudes were derived by averaging across the time window used for beamforming (i.e. 200 to 2000 ms) in the relative and absolute time series data, respectively. Additionally, using these same peak voxel time series data, the envelope of spectral inter-trial phase locking (ITPL) was computed for the time–frequency range used in the beamforming analysis (i.e. 14.5–15.5 Hz; 200–2000 ms) per participant. To evaluate the consistency of visual entrainment amplitudes across trials, the coefficient of variation was calculated across the time/frequency-averaged single-trial response absolute amplitudes for each participant. Finally, to investigate if differences in the baseline-relative amplitude responses were spectrally specific (i.e. restricted to the stimulation frequency), group comparisons were repeated for the relative amplitude responses per frequency bin (i.e. from 4–50 Hz in steps of 0.5 Hz) in the peak visual coordinate during the entrainment time period (i.e. 200–2000 ms). To reduce the impact of outliers on statistical analyses, participants with values 2.5 SDs above or below their respective group mean were excluded for each analysis.

Finally, to assess the internal consistency of our Alzheimer’s disease spectrum group, we conducted follow-up analyses that compared those with aMCI to those with mild Alzheimer’s disease. Briefly, these two Alzheimer’s disease spectrum subgroups were compared on each metric where significant differences between Alzheimer’s disease spectrum and healthy control groups were found (i.e. baseline-relative entrainment amplitude, coefficient of variation and baseline periodic 15 Hz activity).

### Parameterization of baseline activity

Using the same primary visual cortex data as the virtual sensor analyses, we averaged the time–frequency data over the pre-stimulus period (i.e. –600 to 0 ms) to derive baseline power spectral density (PSD) values, per participant. These PSD data were then parameterized using the FOOOF/specparam algorithm, implemented in Python (https://github.com/fooof-tools/fooof). Using this approach, PSDs are separated into oscillatory (i.e. periodic) and non-oscillatory (i.e. aperiodic) signal components.^51^ For the present study, we parameterized the frequency range of 4–50 Hz because this was the frequency range of the virtual sensor analysis and would provide sufficient range for proper fitting of the FOOOF/specparam algorithm.^52^ Spectral parameterization settings for the fitting algorithm were set to defaults (i.e. maximum n_peaks_ = infinite, minimum peak height = 0, peak threshold = 2 SD, fixed aperiodic mode) with peak width limits set to range from 1 to 15 Hz. From the parameterized spectra, baseline absolute amplitude values were obtained by averaging over the frequency range used in the beamforming analysis (i.e. 14.5–15.5 Hz). Finally, goodness of fit values (i.e. R^2^) were extracted per participant to ensure that the quality of parameterization did not differ by group.

### Statistical analyses and software

All statistical analyses were performed using *R*,^53^ and data plots were generated using *ggplot2*.^54^ To complement our initial frequentist statistical approach, Bayesian analysis was also performed in *JASP*,^55^ using a zero-centred Cauchy distribution with a default scale of 0.707. Independent samples *t*-tests were used to test for group differences in average absolute and relative entrainment amplitude, average entrainment ITPL, average entrainment amplitude variability (coefficient of variation), baseline unparameterized 15 Hz activity, baseline periodic 15 Hz activity and baseline aperiodic 15 Hz activity. Linear regression analyses were used to test for relationships between entrainment amplitude and cognitive performance as measured by the neuropsychological testing scores, MMSE, and MoCA.

### Data availability

The data that support the findings of this study are available from the corresponding author, upon reasonable request. Specifically, the anonymized data will be available for non-commercial research purposes and responses will occur at earliest convenience with a goal of delivering the data within 1 month.

The authors assert that all procedures contributing to this work comply with the ethical standards of the relevant national and institutional committees on human experimentation and with the Helsinki Declaration of 1975, as revised in 2008.

## Results

Groups were matched on key demographics except for age (i.e. Alzheimer’s disease spectrum participants were slightly younger). However, inclusion of age as a covariate of no interest in all final statistical models did not change the significance or interpretation of any results. Our results were also unbiased by head movement, as there were no group differences in total head drift (*t*_56_ = 0.32, *P* = 0.754; BF_01_ = 3.46) nor maximum head drift (*t*_56_ = –0.41, *P* = 0.69; BF_01_ = 3.373). Group means and statistical values for comparisons for relevant demographic factors and performance on cognitive testing can be found in Table 1.

### Sensor-level analysis

**Figure 1:**
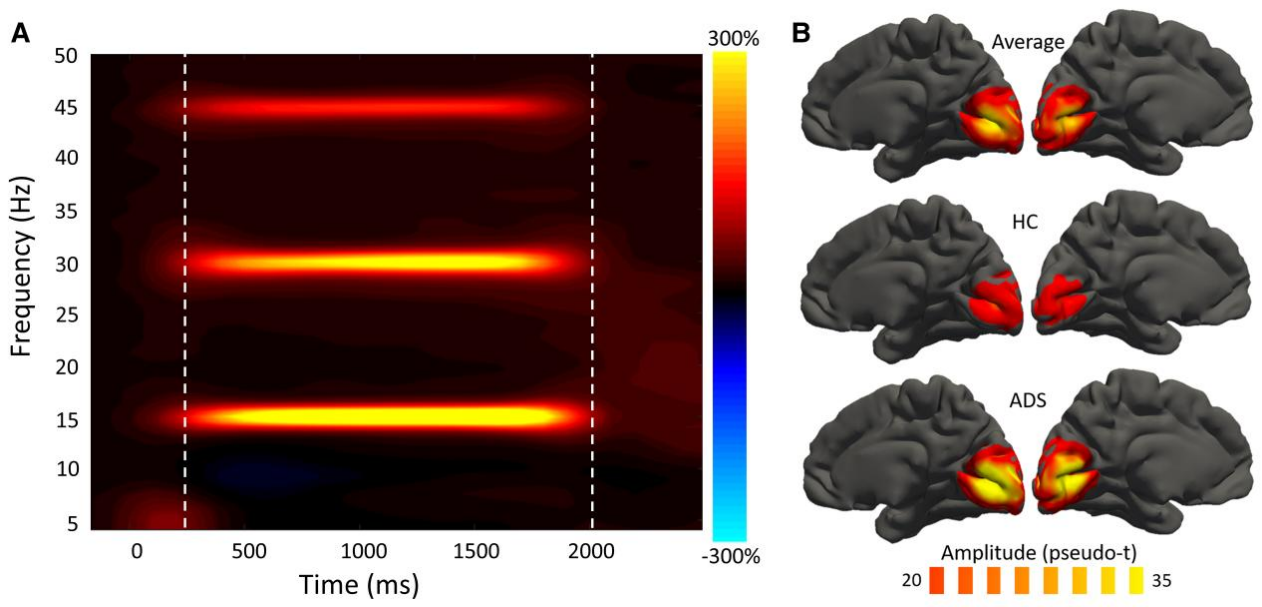
**Sensor- and source-level activity during visual entrainment.** (**A**) Grand-averaged time–frequency spectrogram from a sensor near the occipital cortex (i.e. MEG2343), with time (ms) shown on the *x*-axis and frequency (Hz) denoted on the *y*-axis. A colour scale bar shown to the right of the spectrogram denotes the percent power change relative to the baseline period (−600 to 0 ms). There is clear entrainment to the 15 Hz flicker stimulus and harmonics (i.e. 30 and 45 Hz). (**B**) Mean beamformer images (pseudo-*t*; see colour bar) of the 15 Hz entrainment response (i.e. 200–2000 ms and 14.5–15.5 Hz). ADS = Alzheimer’s disease spectrum, HC = healthy controls.

Sensor-level time–frequency analysis across all participants revealed significant oscillatory responses in a large number of posterior sensors at the base entrainment frequency (i.e. 15 Hz) and harmonics (i.e. 30 and 45 Hz), all of which were increased in amplitude relative to baseline (Fig. 1A). The base frequency entrainment response began around 200 ms after the onset of the entrainment stimulus and lasted until about 500 ms after its removal (i.e. 200–2000 ms; *P* < 0.001, corrected).

### Beamformer and virtual sensor analysis

**Figure 2:**
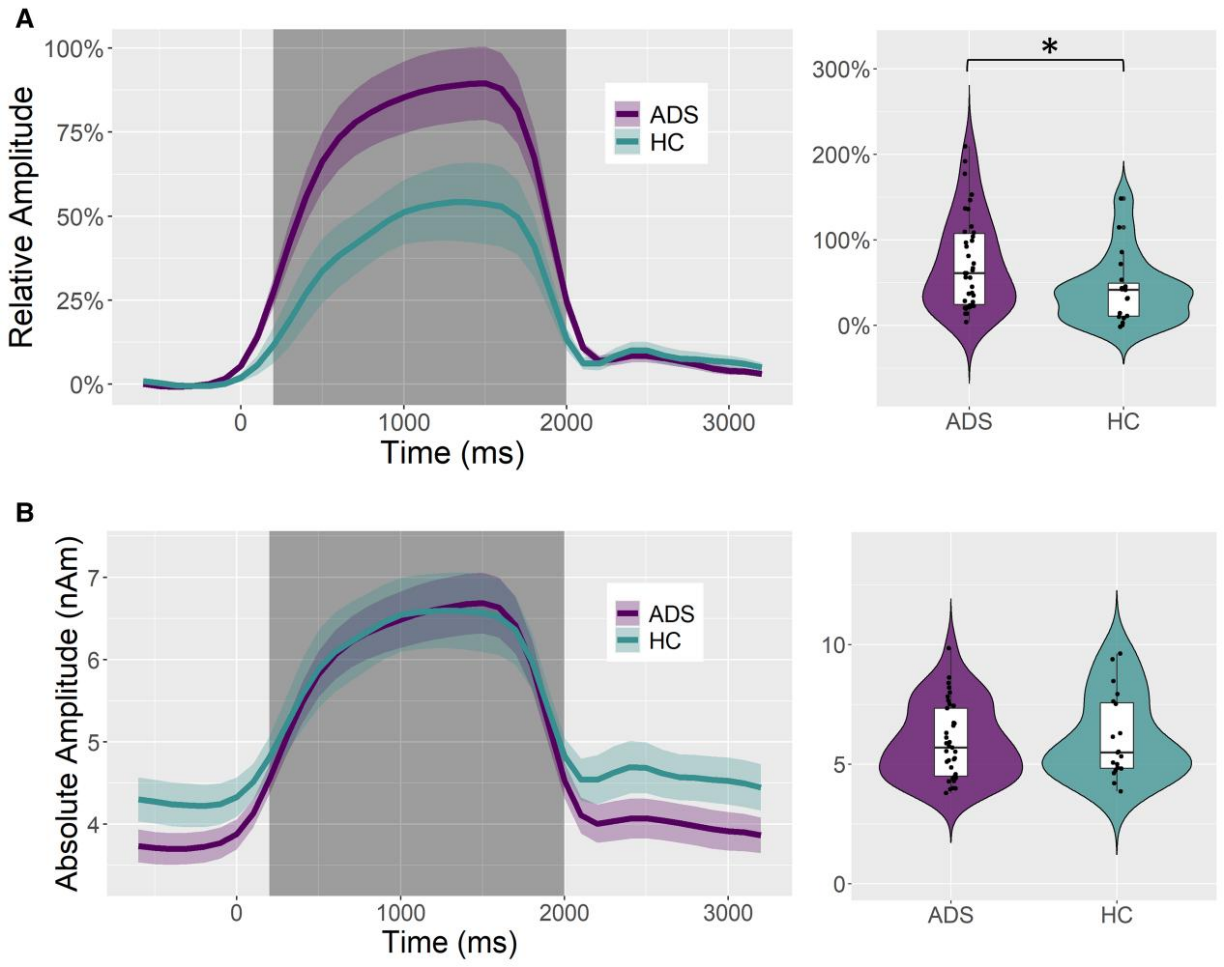
**Absolute and relative amplitude 15 Hz primary visual responses and group differences.** From the peak voxel exhibiting the strongest neural activity in response to the 15 Hz entrainment stimulus, time series were extracted to evaluate differences in entrainment response amplitude as a function of group [healthy controls (HCs); Alzheimer’s disease spectrum (ADS)] during the response time window identified through the sensor-level analysis (i.e. 200–2000 ms; shaded area). Relative time series (i.e. baseline-corrected) are shown in (**A**) and absolute amplitude time series (i.e. not baseline-corrected) are shown in (**B**). Violin plots to the right of each time series plot represent the smoothed probability density of the average entrainment amplitude per subject, separated by group, during the response time window (shaded area). Box plots show group-wise means, first and third quartiles, and minima and maxima *P < 0.05. Group comparisons were made using independent samples *t*-tests (relative: *t*_55_ = –2.21, *P* = 0.031; absolute: *t*_54_ = 0.41, *P* = 0.68).

To determine the cortical regions generating the entrainment response, the previously identified sensor-level time–frequency bin (i.e. 200–2000 ms and 14.5–15.5 Hz) was imaged using a frequency-resolved beamformer. Strong increases in 15 Hz activity were observed from 200–2000 ms in the bilateral primary visual cortices (Fig. 1B). To quantify the spectrotemporal dynamics of this visual entrainment response and evaluate group differences in the processing of the entrainment stimulus, absolute amplitude and baseline-relative virtual sensor time series were computed for the voxel with the greatest 15 Hz response amplitude across all participants (Fig. 2). In both groups, 15 Hz primary visual activity sharply increased during visual stimulation, with greater entrainment responses relative to the pre-stimulus baseline period in patients on the Alzheimer’s disease spectrum compared to healthy controls (*t*_55_ = –2.21, *P* = 0.031; BF_01_ = 0.497; Cohen’s d = 0.621; Fig. 2A). This effect was spectrally specific to the frequency of entrainment (Supplementary Fig. 1). Interestingly, there was no such group difference in the absolute (i.e. baseline-invariant) amplitude of visual entrainment (*t*_54_ = 0.41, *P* = 0.683; BF_01_ = 3.307; Fig. 2B).

**Figure 3:**
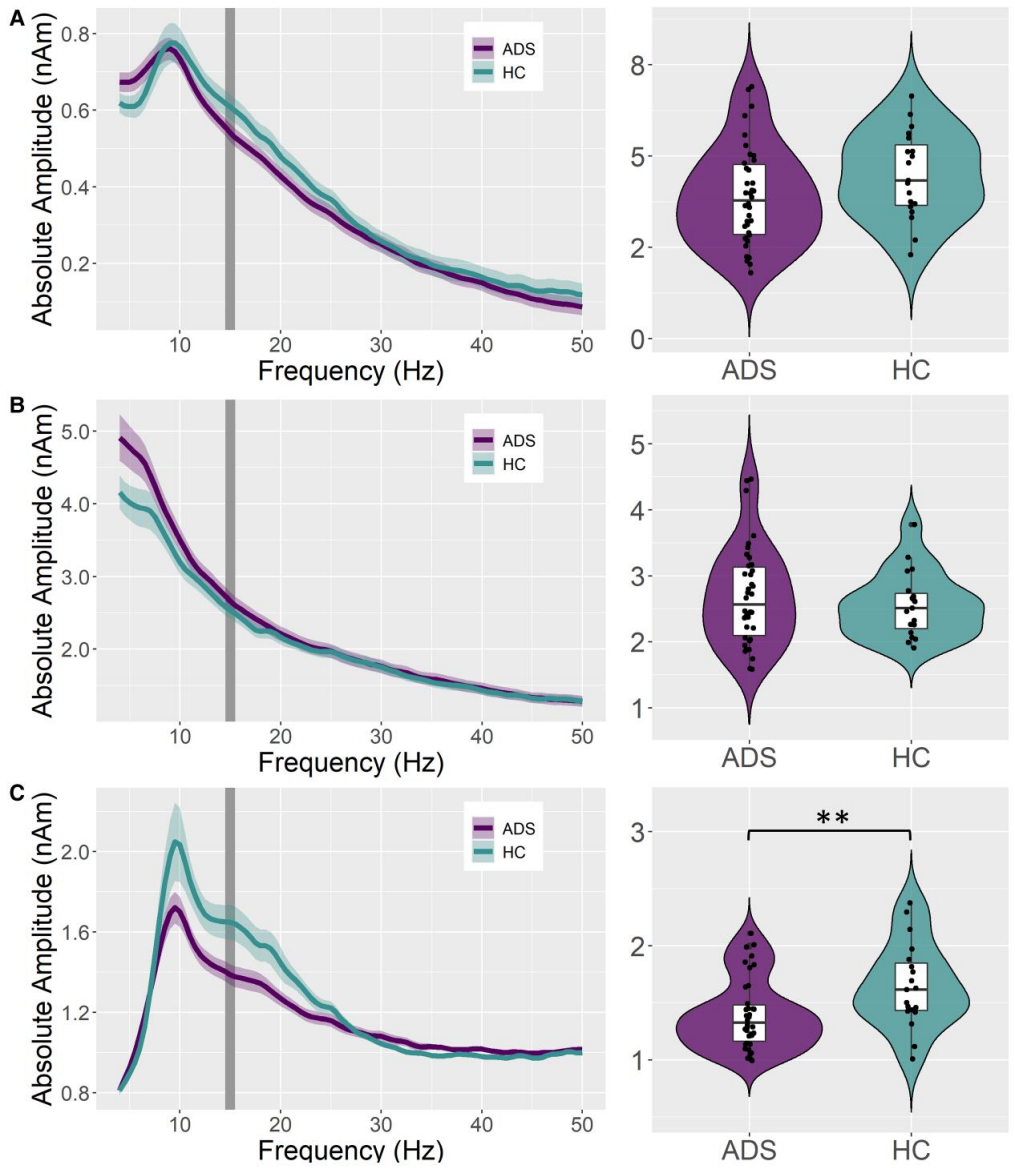
**Absolute 15 Hz baseline activity in primary visual cortices and group differences.** From the peak voxel exhibiting the strongest neural activity in response to the 15 Hz entrainment stimulus, PSDs were extracted across the baseline period (i.e. –600 to 0 ms) to evaluate differences in pre-stimulus 15 Hz activity as a function of group (healthy controls (HC); AD spectrum (ADS) at the entrainment frequency identified through the sensor-level analysis (i.e. 14.5–15.5 Hz; shaded area). The average PSD of the unparameterized signal is shown per group in (**A**), the average PSD of the aperiodic signal component is shown in (**B**), and the PSD of the periodic signal component is shown in (**C**). Violin plots to the right of each PSD plot represent the smoothed probability density of the average entrainment amplitude per subject, separated by group, during the baseline period within the base frequency of entrainment (shaded area). Box plots show group-wise means, first and third quartiles, and minima and maxima **P < 0.01. Group comparisons were made using independent samples *t*-tests (A: *t*_55_ = 1.59, *P* = 0.117; B: *t*_55_ = –0.73, *P* = 0.468; C: *t*_55_ = 2.75, *P* = 0.008).

Having found differences in relative but not absolute entrainment responses, we next investigated if there were group differences in baseline 15 Hz activity. There was no group difference in baseline 15 Hz activity in the unparameterized signal (*t*_55_ = 1.59, *P* = 0.12; BF_01_ = 1.264; Fig. 3A) nor the aperiodic component (*t*_55_ = –0.73, *P* = 0.468; BF_01_ = 2.86; Fig. 3B). However, there was a significant group difference in the periodic component of baseline 15 Hz activity, such that patients on the Alzheimer’s disease spectrum had lower periodic 15 Hz activity during the baseline period compared to healthy controls (*t*_55_ = 2.75, *P* = 0.008; BF_01_ = 0.18; Cohen’s d = 0.773; Fig. 3C). Importantly, there was no group difference in the subject-wise model fit for the parameterization of baseline activity (*t*_56_ = 0.32, *P* = 0.750).

**Figure 4:**
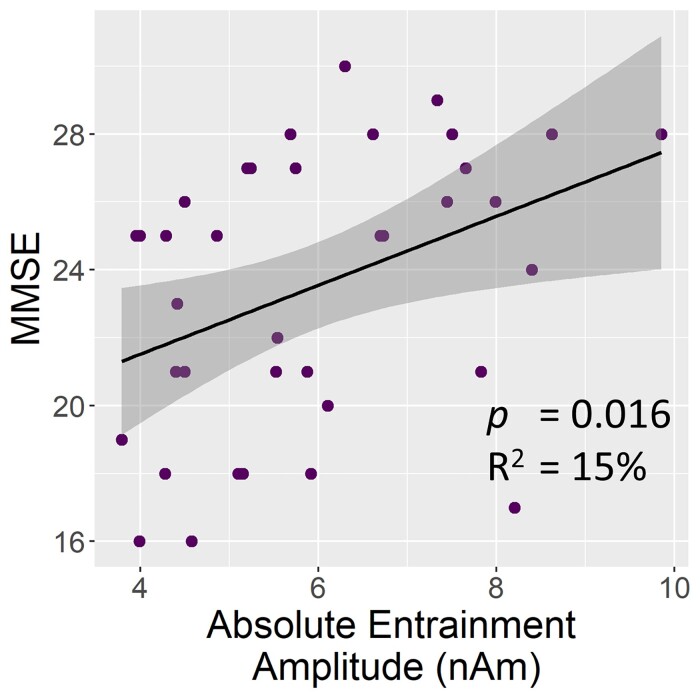
**Relationship between absolute entrainment amplitude and cognitive performance.** The regression plot shows the relationship between general cognitive performance (i.e. MMSE score) and absolute entrainment amplitude in patients on the AD spectrum. Lines of best-fit, 95% CI (shaded area), and relevant statistics are overlaid.

In order to evaluate the relationship between entrainment amplitude and cognitive performance, MMSE scores were regressed on neural entrainment response amplitudes for participants on the Alzheimer’s disease spectrum (see Supplemental Material for a similar regression with MoCA scores). Only the absolute level of visual entrainment (*F*_1,35_ = 6.36, *P* = 0.016; BF_01_ = 0.305; η^2^p = 0.154), and not the relative increase in response amplitude from baseline (*F*_1,36_ = 0.15, *P* = 0.70, BF_01_ = 4.618), predicted cognitive abilities (Fig. 4). Further, composite neuropsychological measures from several cognitive domains (i.e. attention, processing speed, and memory) were separately regressed on neural entrainment response amplitudes for participants on the Alzheimer’s disease spectrum. Neither absolute level of visual entrainment nor relative (i.e. baseline-corrected) response amplitudes predicted neuropsychological performance in the domains of attention (absolute: *F*_1,35_ = 2.40, *P* = 0.130, BF_01_ = 1.624; relative: *F*_1,36_ = 0.382, *P* = 0.540, BF_01_ = 4.133), processing speed (absolute: *F*_1,35_ = 2.39, *P* = 0.131, BF_01_ = 1.632; relative: *F*_1,36_ = 0.02, *P* = 0.886, BF_01_ = 4.902), or memory (absolute: *F*_1,35_ = 3.47, *P* = 0.071, BF_01_ = 1.016; relative: *F*_1,36_ = 0.18, *P* = 0.678, BF_01_ = 4.556).

**Figure 5:**
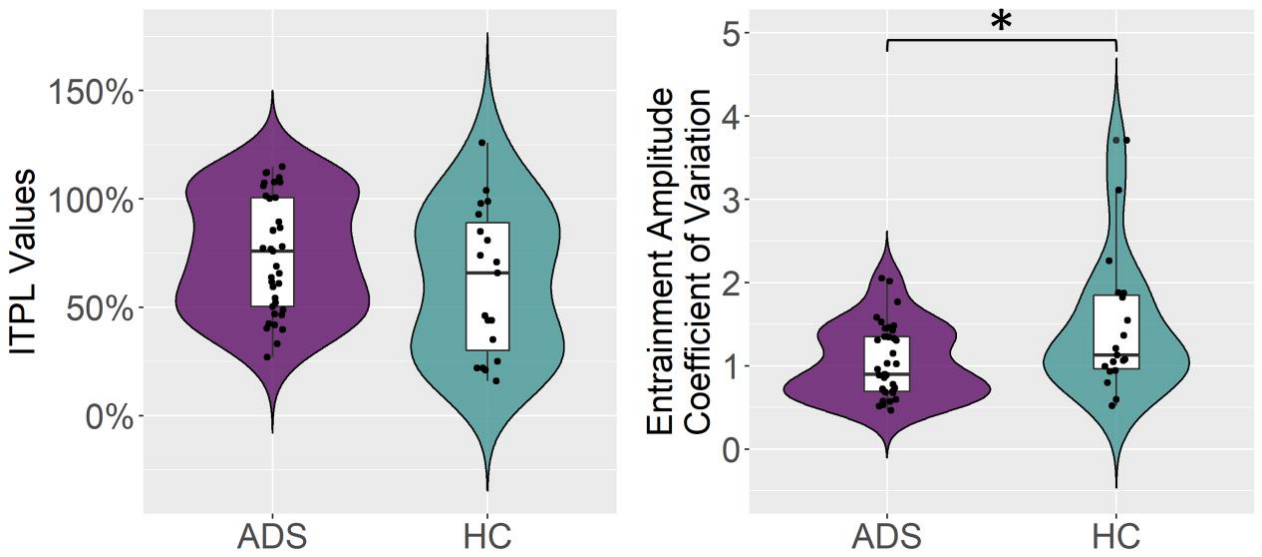
**Entrainment amplitude and phase consistency.** There were no group differences in phase consistency (i.e. inter-trial phase locking; ITPL) across trials, but there were significant group differences in amplitude consistency across trials (i.e. coefficient of variation; CV) such that participants on the AD spectrum had more consistent responses (i.e. lower CV) than healthy controls. Violin plots represent the smoothed probability density of the average single-trial ITPL values (left) and CV (right) per subject, separated by group. Box plots show group-wise means, first and third quartiles, and minima and maxima *P < 0.05. Group comparisons were made using independent samples *t*-tests (ITPL: *t*_56_ = –1.24, *P* = 0.22; CV: *t*_54_ = 2.50, *P* = 0.015).

To probe the origins of these differences, we examined the consistency of the entrainment phase and amplitude across trials (Fig. 5). We found no group difference in the phase consistency (i.e. ITPL) during the 200–2000 ms interval (*t*_56_ = –1.24, *P* = 0.22; BF_01_ = 1.927). In contrast, group differences in the consistency of the response amplitude across trials (i.e. coefficient of variation) were detected such that patients on the Alzheimer’s disease spectrum had more consistent cross-trial entrainment amplitudes than healthy controls (*t*_54_ = 2.50, *P* = 0.015; BF_01_ = 0.290; Cohen’s d = 0.707).

Lastly, to ensure the internal consistency of our Alzheimer’s disease spectrum group, we probed for differences between the two Alzheimer’s disease spectrum subgroups (i.e. aMCI and mild probable Alzheimer’s disease) on all significant metrics found in the previous group contrasts. We found no significant differences in 15 Hz relative entrainment responses (*t*_36_ = 0.15, *P* = 0.881; BF_01_ = 3.132), consistency of the response amplitude across trials (*t*_35_ = 0.78, *P* = 0.438; BF_01_ = 2.463), or baseline periodic 15 Hz activity (*t*_36_ = 0.91, *P* = 0.371; BF_01_ = 3.366) in patients with aMCI versus those with mild probable Alzheimer’s disease. Additionally, there was no Alzheimer’s disease spectrum subgroup-by-absolute amplitude entrainment response interaction on MMSE score (MMSE: *F*_3,33_ = 0.718, *P* = 0.403).

## Discussion

Recent animal research has sparked interest in rhythmic visual entrainment of neuronal populations in patients with Alzheimer’s disease. However, to date, there have been no neuroimaging studies aimed at examining the integrity of cortical entrainment to rhythmic visual stimuli in these patients. This is surprising, considering the utility of sensory entrainment paradigms in probing normal cognitive processes^56–60^ and neural processing in neurological and psychiatric disorders.^21–24^ Further, the recent studies mentioned above involving mouse models of Alzheimer’s disease have shown that sensory entrainment might have clinical utility for patients on the Alzheimer’s disease spectrum, in terms of reducing deposits of pathological proteins and improving cognitive function.^16–18^ The first human study that utilized this therapeutic model of sensory entrainment in patients with Alzheimer’s disease showed stronger functional connectivity in the default mode network and altered cytokines and immune factors following eight weeks of daily entrainment.^61^ Unfortunately, this study was not powered to detect changes in CSF Aβ_42_, total tau, or phosphorylated tau.

In the current study, we found altered primary visual entrainment responses in patients on the Alzheimer’s disease spectrum when compared to healthy controls. Interestingly, absolute levels of neural activity during entrainment were also found to predict cognitive performance (i.e. MMSE), such that patients on the Alzheimer’s disease spectrum with entrainment responses that more closely resembled those of controls had better cognitive performance. Below, these findings and their implications are discussed.

In response to the 15 Hz visual entrainment stimulus, patients on the Alzheimer’s disease spectrum were found to have greater *baseline-relative* amplitude responses compared with healthy controls. However, these Alzheimer’s disease spectrum patients were also, on average, reaching similar levels of *absolute* visual entrainment as controls. Though there were no group differences in baseline 15 Hz activity for the unparameterized neural signal or for the aperiodic signal, there was a strong decrease in the 15 Hz periodic baseline activity in patients on the Alzheimer’s disease spectrum relative to controls. This effect was masked by the aperiodic component of the signal in the unparameterized data, indicating the high utility of spectral parameterization for this study. The observed pattern of relative versus absolute entrainment responses, coupled with decreased periodic baseline activity, indicates that participants on the Alzheimer’s disease spectrum were compensating with stronger entrainment responses to reach the same absolute level of visual entrainment as cognitively normal adults. Further supporting this compensation hypothesis, patients on the Alzheimer’s disease spectrum who had greater visual entrainment responses (i.e. absolute amplitudes closer to the healthy control group) were found to have better general cognitive function, as measured by the MMSE. This indicates that the stronger visual neural responses previously reported in patients with Alzheimer’s disease^10,15^ may not necessarily represent pathology, and instead might be a marker of cognitive compensation. Accordingly, visual entrainment paradigms may represent a particularly useful model of cognitive compensation and/or reserve in patients on the Alzheimer’s disease spectrum, given their relative simplicity and ease of implementation.

Investigation of the ITPL and amplitude (i.e. coefficient of variation) consistency of these visual entrainment responses across groups yielded interesting findings as well. The use of ITPL provides information regarding trial-to-trial phase response consistency that is interpretationally distinct from the coefficient of variation computed on oscillatory amplitude across trials. Specifically, ITPL tells us how consistently the phases of the neural signal are aligning from trial to trial for each participant, while the coefficient of variation is based on oscillatory amplitude metrics reflecting how consistent the strength of the response is from trial to trial. In our study there were no differences in the phase-locking of neural populations to the entrainment stimulus across groups, but patients on the Alzheimer’s disease spectrum exhibited more consistent response amplitudes across trials. Taken together, this indicates that the more consistent visual response amplitudes seen in patients on the Alzheimer’s disease spectrum were likely responsible for the observed pattern of greater overall relative entrainment. Importantly, cognitive and neural variability/flexibility is often beneficial in some situations but detrimental in others,^62^ indicating that this pattern of decreased variability and enhanced entrainment in patients on the Alzheimer’s disease spectrum might only be compensatory under specific conditions. Thus, future research into the relationships between these neural dynamics and domain-specific cognitive declines in Alzheimer’s disease would be useful.

While there are no previous visual entrainment studies in patients with Alzheimer’s disease to which our results can be compared, our findings coincide with those of Yener *et al.*,^15^ in which patients were found to have greater visual responses to a basic light stimulus. Similar to Yener and colleagues, we found greater baseline-relative responses to visual stimulation, and our study extends this finding to rhythmic entrainment stimuli and demonstrates that this effect ultimately leads to comparable absolute visual response amplitudes and is likely compensatory in nature. In contrast, early visual component amplitude decreases and response latency increases have been found in studies of visual evoked potentials in patients on the Alzheimer’s disease spectrum.^8–13^ We believe that these discrepancies are related to the fact that the analysis of visual evoked potentials involves the time-domain averaging of visual responses, leading to the findings being dominated by lower frequencies (e.g. delta band), an interpretation that is also supported by Yener et al.^15^ In contrast, our findings are frequency-resolved and are inherently focused at the base stimulation frequency of 15 Hz, however, future studies should more clearly parse the potential frequency specificity of these findings. Of note, our supplemental analyses suggested that the effect may be specific to the stimulation frequency, at least in the context of entrainment.

Before closing, it is important to note the limitations of this study. For one, no behavioural or eye-tracking data were recorded from participants during task performance, and as such, we were unable to link these neural dynamics to real-time cognition or rule out the impact of more eye movements in one group. Future research integrating visual entrainment stimuli with established cognitive tasks might be useful in more directly elucidating the functional role of these neural changes in patients with Alzheimer’s disease. Regarding eye movement, we would hypothesize that patients with Alzheimer’s disease would disengage from the stimulus more often leading to a decrease in response amplitude; thus, given our pattern of results (i.e. stronger relative responses in patients) we do not feel this is a concern. Additionally, the current study only examined a single stimulation frequency, which, as discussed above, should be investigated further in future studies of patients on the Alzheimer’s disease spectrum. Relatedly, a major future direction for this line of research is comparing the strength of entrainment responses at frequencies that more closely align with those that have been shown to clear Alzheimer’s disease-related pathological proteins (i.e. amyloid and tau) in mouse models of Alzheimer’s disease (e.g. 40 Hz),^16–18^ as well as others that are commonly reported to exhibit spontaneous aberrations in the neural data of patients with Alzheimer’s disease (e.g. slower activity in the delta and theta bands).^63–65^ This is of particular importance, given that these animal studies showing the clearance of amyloid and tau through the use of gamma stimulation and entrainment have yet to be replicated in human patients living with Alzheimer’s disease. Of note, while we found clear relationships between the strength of absolute neural entrainment and MMSE in patients on the Alzheimer’s disease spectrum, we found no significant relationships between specific neuropsychological domains and entrainment. However, this is perhaps unsurprising, as the tests within each domain did not explicitly target visual and visuospatial abilities, which are the most likely to relate to the neural responses in this study. Thus, specific neuropsychological analysis of visual and visuospatial abilities would have been a valuable addition to this study and should be considered in future work. Due to concerns over cost and participant risk, we did not collect tau PET data from our patient group, which could have provided novel information regarding the relationship between neurophysiological alterations and proteinopathy in Alzheimer’s disease. Lastly, while our patient sample included participants across a wide range of the Alzheimer’s disease spectrum (i.e. from amnestic MCI to mild Alzheimer’s disease), extending our analyses to patients in the pre-clinical stages of Alzheimer’s disease and to atypical phenotypes (i.e. behavioural/dysexecutive variant, posterior cortical atrophy, etc.) is a critical next step in furthering our understanding of the pathophysiology of the disease as a whole. Translating this line of research to more clinically relevant avenues may unveil more optimal treatment options to improve the lives of patients with Alzheimer’s disease.

## Supplementary Material

fcac198_Supplementary_Data

## Abbreviations

ADS =: Alzheimer’s disease spectrum
aMCI =: amnestic mild cognitive impairment
Aβ =: Amyloid beta
BESA =: Brain Electrical Source Analysis
FIR =: Finite impulse response
FOOOF =: fitting oscillations & one over f
HC =: healthy controls
HVLT-R =: Hopkins Verbal Learning Test-Revised
ITPL =: Inter-trial phase locking
MCI =: mild cognitive impairment
MEG =: magnetoencephalography
MMSE =: Mini-Mental Status Examination
MoCA =: Montreal cognitive assessment
PSD =: power spectral density
WAIS-IV =: Wechsler Adult Intelligence Scale
WMS-IV =: Wechsler Memory Scale.
