# Supplementary material for: Altered visual entrainment in patients with Alzheimer’s disease: magnetoencephalography evidence
Source: Brain Commun. 2022 Aug 1;4(4):fcac198. doi: 10.1093/braincomms/fcac198 (PMC9374481; doi:10.1093/braincomms/fcac198)
Supplement: fcac198_Supplementary_Data [file fcac198_supplementary_data.docx]

***Manuscript Title:*** ***Altered Visual Entrainment in Patients with Alzheimer’s Disease: MEG Evidence***

**Authors:** Seth D. Springer, Alex I. Wiesman, Pamela E. May, Mikki Schantell, Hallie J. Johnson, Madelyn P. Willett, Camilo A. Castelblanco, Jacob A. Eastman, Nicholas J. Christopher-Hayes, Sara L. Wolfson, Craig M. Johnson, Daniel L. Murman, Tony W. Wilson

# Supplementary Material


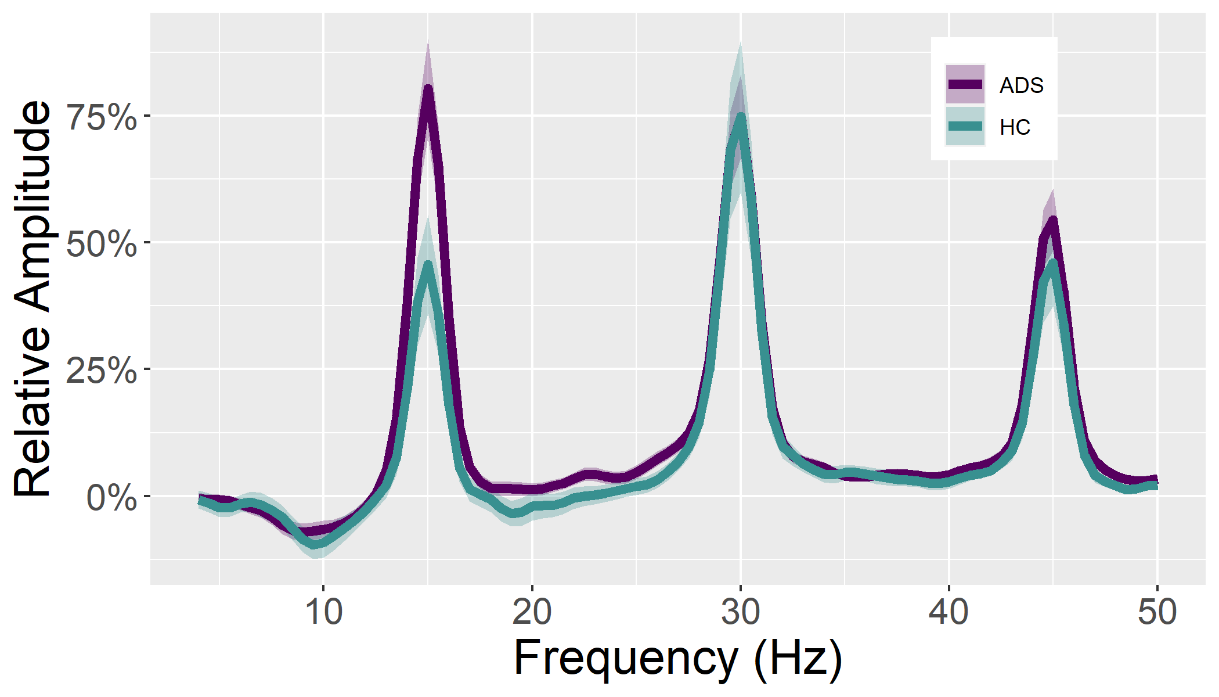


**Supplementary Figure 1.** **Broadband group comparisons of** **relative entrainment amplitude.** Frequency spectra were extracted from the peak voxel exhibiting the strongest neural activity in response to the 15 Hz entrainment stimulus (i.e., 200 to 2000 ms), to evaluate differences in relative (i.e., baseline-corrected) entrainment response amplitude across the broadband as a function of group (healthy controls (HC): teal; AD spectrum (ADS): purple).

**Montreal Cognitive Assessment (MoCA) scores and Entrainment**

In addition to regressing MMSE scores on neural entrainment response amplitudes in participants on the AD spectrum, the same was done with MoCA scores. Similar to the results for the MMSE scores, only the absolute level of visual entrainment (*F*_1,31_ = 6.67, *p* = .015; BF_01_ = 0.268; η^2^p = .177), and not the relative increase in response amplitude from baseline (*F*_1,32_ = 0.1, *p* = .76, BF_01_ = 4.480), predicted cognitive abilities (Supplementary Figure 2). Additionally, there was no AD spectrum subgroup-by-absolute entrainment amplitude interaction on cognitive function (*F*_3,29_ = 0.854, *p* = .363).


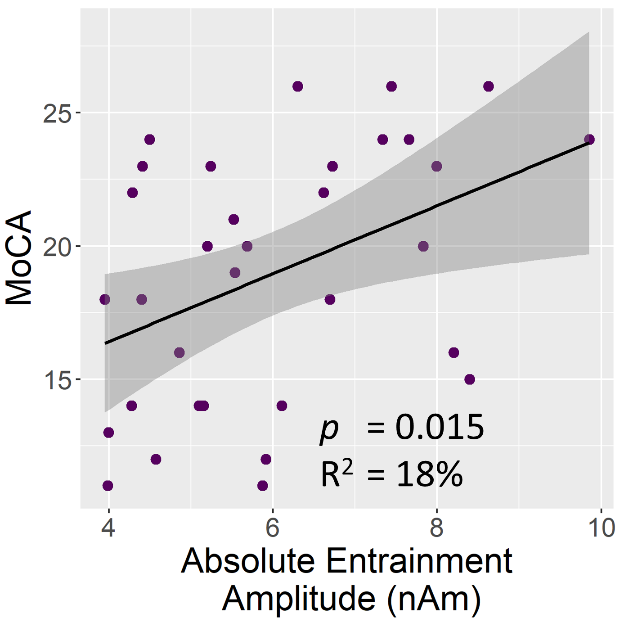


**Supplementary Figure 2. Relationship between absolute entrainment amplitude and MoCA performance.** The regression plot shows the relationship between general cognitive performance (i.e., MoCA score) and absolute entrainment amplitude in patients on the AD spectrum. Lines of best-fit, 95% CI (shaded area), and relevant statistics are overlaid.
